# Supplementary material for: Identification of photoperiod-induced specific miRNAs in the adrenal glands of Sunite sheep (Ovis aries)
Source: Front Vet Sci. 2022 Jul 22;9:888207. doi: 10.3389/fvets.2022.888207 (PMC9354845; doi:10.3389/fvets.2022.888207)
Supplement: Supplementary file 1 [file Data_Sheet_1.ZIP › Supplementary file/miRDeep2 file.zip!/miRDeep2 file/Know_Structure/SPLP42A2/oar-let-7f.pdf]

miRBase precursor : oar-let-7f
Total read count : 642461
oar-let-7f read count : 642343
remaining reads : 118

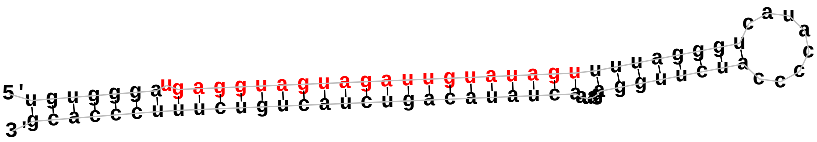

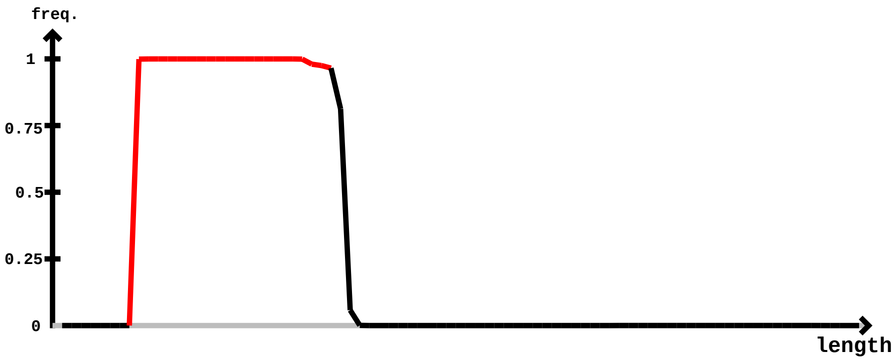


```
           oar-let-7f
5'- uguggga ugagguaguagauuguauagu uuuagggucauaccccaucuuggagauaacuauacagucuacugucuuucccacg   -3'    exp
    .(((((.(((((((((((((((((((((((((((((........))))))))....)))))))))))))))))))))))))).    reads    mm    sample
    ......augagguaguagauuguau..........................................................    1        0     seq
    ......augagguaguagauuguauagu.......................................................    10       0     seq
    ......Gugagguaguagauuguauagu.......................................................    1        1     seq
    ......augagguaguagauuguauaguu......................................................    17       0     seq
    ......Gugagguaguagauuguauaguu......................................................    5        1     seq
    ......augagguaguagauuguauaguuu.....................................................    2        0     seq
    .......ugaAguaguagauugua...........................................................    2        1     seq
    .......ugagguGguagauugua...........................................................    2        1     seq
    .......ugagguaguagauugua...........................................................    466      0     seq
    .......ugagguaguagauugAa...........................................................    1        1     seq
    .......ugagguaguUgauugua...........................................................    2        1     seq
    .......ugagAuaguagauugua...........................................................    6        1     seq
    .......ugagguaguagauuguU...........................................................    10       1     seq
    .......ugagguaguagauuguUu..........................................................    8        1     seq
    .......ugaAguaguagauuguau..........................................................    87       1     seq
    .......ugagguaguagauuguau..........................................................    11984    0     seq
    .......ugagguaguUgauuguau..........................................................    18       1     seq
    .......ugagguaguagauugAau..........................................................    3        1     seq
    .......ugagguaguagauuguaua.........................................................    3358     0     seq
    .......ugagguaguagauugAaua.........................................................    1        1     seq
    .......ugagguaguagauuguauag........................................................    6142     0     seq
    .......ugagguaguagauuguauagu.......................................................    98182    0     seq
    .......ugagguaguagauugAauagu.......................................................    16       1     seq
    .......ugagguaguagauuguauaguu......................................................    484569   0     seq
    .......ugagguaguagauuguauaguuu.....................................................    34018    0     seq
    .......ugagguaguagauuguauaguuG.....................................................    2590     1     seq
    .......ugagguaguagauuguauaguuGu....................................................    103      1     seq
    .......ugagguaguagauuguauaguuuu....................................................    307      0     seq
    .......ugagguaguagauuguauaguuuua...................................................    2        0     seq
    ........gagguaguagauuguau..........................................................    1        0     seq
    ........gagguaguagauuguaua.........................................................    2        0     seq
    ........gagguaguagauuguauag........................................................    3        0     seq
    ........gagguaguagauuguauagu.......................................................    52       0     seq
    ........gagguaguagauuguauaguu......................................................    290      0     seq
    ........gagguaguagauuguauaguuG.....................................................    2        1     seq
```

oar-let-7f

```
uguggga**ugagguaguagauuguauagu**uuuagggucauaccccaucuuggagauaacuauacagucuacugucuuucccacg
........gagguaguagauuguauaguuu.....................................................    21    0    seq
.........agguaguagauuguauagu.......................................................    1     0    seq
.........agguaguagauuguauaguu......................................................    4     0    seq
.........agguaguagauuguauaguuu.....................................................    3     0    seq
.........agguaguagauuguauaguuG.....................................................    4     1    seq
.........agguaguagauuguauaguuGu....................................................    39    1    seq
..........gguaguagauuguauagu.......................................................    1     0    seq
..........gguaguagauuguauaguu......................................................    5     0    seq
..........gguaguagauuguauaguuG.....................................................    1     1    seq
............uaguagauuguauaguu......................................................    1     0    seq
.............................uuagggucauaccccaucuugga...............................    2     0    seq
.............................uuagggucauaccccaucuuggaga.............................    2     0    seq
.......................................................aacuauacagucuacugucu........    1     0    seq
.........................................................cuauacagucuacugucuuu......    8     0    seq
.........................................................cuauacagucuacugucuuuc.....    19    0    seq
.........................................................cuauacagucuacugucuuucc....    86    0    seq
```
